# Control of chronic excessive alcohol drinking by genetic manipulation of the Edinger–Westphal nucleus urocortin-1 neuropeptide system

**DOI:** 10.1038/tp.2016.293

**Published:** 2017-01-31

**Authors:** W J Giardino, E D Rodriguez, M L Smith, M M Ford, D Galili, S H Mitchell, A Chen, A E Ryabinin

**Affiliations:** 1 Department of Behavioral Neuroscience, Oregon Health & Science University, Portland, OR, USA; 2 Department of Neurobiology, Weizmann Institute of Science, Rehovot, Israel; 3 Department of Stress Neurobiology and Neurogenetics, Max Planck Institute of Psychiatry, Munich, Germany

## Abstract

Midbrain neurons of the centrally projecting Edinger–Westphal nucleus (EWcp) are activated by alcohol, and enriched with stress-responsive neuropeptide modulators (including the paralog of corticotropin-releasing factor, urocortin-1). Evidence suggests that EWcp neurons promote behavioral processes for alcohol-seeking and consumption, but a definitive role for these cells remains elusive. Here we combined targeted viral manipulations and gene array profiling of EWcp neurons with mass behavioral phenotyping in C57BL/6 J mice to directly define the links between EWcp-specific urocortin-1 expression and voluntary binge alcohol intake, demonstrating a specific importance for EWcp urocortin-1 activity in escalation of alcohol intake.

## Introduction

Alcoholism can emerge as a maladaptive coping strategy in stress-experienced individuals, highlighting the importance of biological stress systems in maintaining addiction.^1^ Stress-responsive neuronal populations release peptide modulators to facilitate appropriate responding to environmental challenges, but dysregulation of these circuits can have profound consequences on behavior.^2, 3^ Following repeated cycles of alcohol intoxication and withdrawal, stress-sensitive neural systems can perpetuate the addiction cycle via persistent adaptations known as allostatic shifts.^4, 5^ In particular, recent investigations of stress-related neuropeptide contributions to binge alcohol drinking focused on the bed nuclei of stria terminalis,^6^ lateral hypothalamus,^7^ central nucleus of the amygdala,^8, 9, 10, 11^ ventral tegmental area,^12, 13, 14^ and dorsal raphe nucleus.^15, 16^ These are well-established regions in the addiction neural network, and likely interact at the circuit level with other uncharacterized neuronal populations to coordinate complex motivated behaviors.

Here, we focused on the neuronal population within the ventromedial periaqueductal gray that comprises the centrally projecting Edinger–Westphal nucleus (EWcp).^17^ EWcp neurons are proposed to interact with regions of the classic stress and addiction neurocircuits described above,^16, 18, 19, 20, 21, 22, 23, 24^ as well as the lateral septum, brainstem and spinal cord.^17, 25, 26, 27, 28, 29, 30^ EWcp neurons are activated by stressors^31^ and highly enriched in genes encoding neuropeptide transmitters: cocaine- and amphetamine-related transcript (*Cart*);, cholecystokinin (*Cck*); nesfatin-1 (*Nucb2*); pituitary adenylyl cyclase-activating polypeptide (PACAP, *Adcyap1*); and urocortin-1 (Ucn1, *Ucn*).^32^ Although each of these neuropeptides are appealing candidates to pursue, Ucn1 is one of four endogenous ligands of the corticotropin-releasing factor (CRF) family, and an extensive literature implicates the CRF/urocortin system in facilitating and maintaining drug addiction.^3, 22, 23, 33^

The intricate organization of the endogenous CRF system permits several possible pharmacological interactions. For example, Ucn1 displays high-affinity interactions with both G-protein-coupled CRF receptors, as well as the CRF binding protein).^34^ Consequently, the precise anatomical origins of the CRF system ligands underlying addiction-related traits remain largely undefined.^35^ For example, rather than manipulating endogenous CRF peptide-containing cell body populations, many CRF-related alcohol studies focused on receptor mechanisms and supraphysiological administration of exogenous pharmacological agents.^36^ As the EWcp is the primary endogenous site of Ucn1 expression in the mammalian brain, we performed targeted genetic perturbations of endogenous EWcp-Ucn1 activity to demonstrate a selective role for this neuronal population in heavy alcohol drinking. In complementary experiments, we characterized EWcp molecular adaptations following long-term free-choice intermittent binge alcohol drinking, and report novel data on dynamic changes in multiple stress-related neuropeptide system genes. Altogether, our findings establish the EWcp as a fundamental component of the alcohol addiction neurocircuitry.

## Materials and methods

### Animals

C57BL/6 J (B6) mice inherently prefer alcohol and display patterns of voluntary consumption that surpass the criterion for ‘binge drinking' (blood alcohol concentrations >80 mg dl^−1^ (BACs, >0.08%)), providing a genetically tractable model for elucidating the underpinnings of excessive alcohol intake.^37^ In all current studies, mice had 24 h access to water and standard chow diet. Extensive analyses of consummatory-related control variables were used to dissociate motivational factors underlying alcohol-seeking and consumption from thirst, taste, and caloric drive.^38^

For studies using adult male and female Ucn1 knockout (KO) and wild-type (WT) littermate mice, animals were originally generated on a 129X1/SvJ x B6 background containing a deletion of exon 2 of the *Ucn* gene,^39^ and then backcrossed to B6 for 10–12 generations.^40^ KO and WT mice were littermates generated by heterozygous matings, weaned at 21–28 days of age, and isosexually housed. For studies in male B6 mice, 8-week-old animals were ordered from The Jackson Laboratory (Sacramento, CA, USA).

### General procedures

Mice were initially housed four per cage in a temperature- and humidity-controlled environment with *ad libitum* access to food (LabDiet 5001; LabDiet, Richmond, IN, USA) and water on a 12/12 light/dark cycle with lights ON at 0600 hours. Prior to drinking studies, all mice were transferred to the procedure room (lights OFF at 0800 h), and allowed to acclimate to single housing and the reverse light–dark cycle for 7 to 14 days. During acclimation, mice received access to two 25 ml glass cylinder bottles with metal sipper tubes (both containing water) on either side of the cage, with food evenly distributed across the cage top. Measurements of body weight, water, and food consumption during acclimation allowed calculation of baseline consummatory variables. The side of the alcohol or tastant bottle was fully counterbalanced across groups. Furthermore, the side of the alcohol or tastant bottle was regularly switched to avoid the potential confound of a side bias. Water-only control mice experienced identical bottle configurations and patterns of bottle switching. Alcohol (95%) was diluted in tap water to the desired concentrations and expressed in volume/volume units. Protocols were approved by the Oregon Health & Science University Institutional Animal Care and Use Committee, performed with adherence to the NIH Guidelines for the Care and Use of Laboratory Animals.

For alcohol drinking studies, primary variables of interest included: alcohol intake (grams alcohol consumed per kilograms body weight (g kg^−1^)), alcohol preference ratio (volume alcohol consumed/volume total fluid consumed), total fluid intake (ml of water+alcohol consumed), food intake (g food consumed per kg body weight), and total caloric intake (calories consumed from 5001 rodent chow+calories consumed from alcohol), as in Giardino *et al.*^38^ Thus, we incorporated analyses of multiple consummatory-related variables in order to dissociate motivation for alcohol's pharmacological properties vs generalized thirst- or calorie-driven factors. Multi-factor analyses of variance (ANOVAs) initially included sex as a factor, combining sexes only in the absence of statistically significant interactions between sex and genotype.

### Effects of Ucn1 KO on escalating continuous access drinking

Male and female Ucn1 KO and WT mice (*n*=7–14 per sex, per genotype) received 24 h two-bottle choice access to increasing concentrations of alcohol for twelve consecutive days (10, 20 and 40%, four days at each concentration), similar to previously described studies from our group.^20^ Sex × genotype interactions for alcohol intake, alcohol preference and total fluid intake failed to reach significance (F_1,40_=3.82, 0.12, 2.75; *P*=0.06, 0.73, 0.11), thus sexes were combined for analyses. Data were analyzed by two-way repeated measures (RM)-ANOVA, using genotype as the between-subjects factor and alcohol concentration as the repeated measure. *Post hoc* Bonferroni comparisons were made following significant (*P*<0.05) genotype interactions.

### Effects of Ucn1 KO on non-escalating continuous access drinking

Male and female Ucn1 KO and WT mice were used (*n*=4–9 per sex, per genotype). On the first day, following habituation to the reverse light–dark cycle (Day 1), at 2 h into the dark cycle, water in one of the two water bottles was replaced with 10% alcohol and remained on the cage top for 12 consecutive days. Bottles were read daily at 2 h into the dark cycle. Twelve days of 10% continuous access were divided into 4-day ‘bins' (Days 1–4, 5–8, 9–12) for comparison of analyses to those of the previous experiment. Sex × genotype interactions for 24 h alcohol intake, alcohol preference and total fluid intake failed to reach statistical significance (F_1,22_=0.01, 0.32, 0.20; *P*=0.92, 0.58, 0.66), thus sexes were combined for all analyses. Two-way RM-ANOVA was used to analyze the data (between-subjects factor of genotype, repeated measure of bin).

### Effects of Ucn1 KO on escalating intermittent access drinking

This experiment was performed using a lickometer system (MedPC; Med Associates, St. Albans, VT, USA). Mice were housed in small Plexiglas cages on top of a metal grid floor, with alligator clips attached to wires connecting the grid floor to the metal spouts of the bottles. Each lick (with 10 ms resolution) was recorded by completion of an electrical circuit, and data were stored on an interfaced PC computer. Ucn1 KO and WT mice (*n*=5–9 per sex, per genotype) were used. Mice underwent the long-term intermittent access procedure, receiving every-other-day 22 h access sessions with alcohol offered at increasing concentrations of 3, 6, and 10% on Days 1, 3 and 5, respectively, followed by 20% every-other-day for 1 month. Two hours into the dark cycle, alcohol bottles were weighed, and one of two water bottles on each cage was replaced with a bottle containing alcohol. Twenty-two hours later, the alcohol bottles were removed and replaced with the original additional water bottles. With each exchange, bottles were weighed to the nearest 0.1 g. An alcohol bout was defined as twenty or more consecutive alcohol licks, with each lick separated by <1 min. Several alcohol bout variables were generated from the data and used to analyze the alcohol-drinking microstructure (bout frequency, bout size, interbout interval, bout length, bout rate and latency to first bout).

Sex × genotype interaction for alcohol licks failed to reach statistical significance (F_1,25_=0.00; *P*=0.96), thus sexes were combined for all analyses. In order to validate the lickometer apparatus, mean alcohol intake and alcohol lick data were averaged across all 20% alcohol sessions and analyzed by Pearson's correlation. To investigate differential *patterns* of 20% alcohol drinking between the genotypes, alcohol lick data across 20% alcohol days were analyzed by linear regression models.

To visualize the circadian timecourse of alcohol licks, hourly data from 20% alcohol sessions (Day 7 to Day 35) were averaged and plotted, allowing determination that genotype differences were greatest during the initial 4 h of each drinking session. This 4 h interval corresponded to the timeperiod of drinking in the standard ‘drinking-in-the-dark' model, thereby facilitating comparison of results between studies. Four-hour alcohol bout data were analyzed across 20% alcohol-drinking sessions by linear regression. Alcohol licks collected during the final 4 h drinking session on Day 37 were compared between genotypes by *t*-test. Day 37 blood alcohol concentrations (BACs) measured by gas chromatography (GC) and Analox were analyzed by Pearson's correlation to determine between-method reliability, and then subjected to RM-ANOVA (between-subjects factor of genotype, repeated measure of method (GC vs Analox)).

### Effects of long-term intermittent alcohol drinking on EWcp gene expression

Following habituation, 8-week-old male B6 mice were randomly split into groups receiving access to Alcohol or H_2_O only (Naive Controls). Mice underwent the long-term intermittent access procedure as described above (or underwent the procedure in parallel, except as alcohol-naive water-only controls). Food was regularly collected and weighed from the cage tops at the beginning and end of sessions. During food weighing, cages were checked for pieces of food pellets that fell through the cage top (<0.10% of all food measurements).

At the beginning of Day 37 of the long-term intermittent access paradigm, mice were killed by carbon dioxide (CO_2_) at ZT-14 (zeitgeiber time; lights OFF at ZT-12), which is immediately prior to the time at which they would have normally next received alcohol access. Thus, Alcohol mice experienced 24 h of forced abstinence at the time of euthanasia. Brains were dissected, and tissue samples containing the EWcp from each animal were dissected prior to undergoing RNA extraction, isolation, and quantification as detailed below.

### Gene expression analyses

EWcp gene expression studies were conducted similarly to previous publications from our group.^32, 41^ Dissected brains were immediately placed inside a pre-chilled coronal brain matrix. A 1-mm-thick tissue punch containing the EWcp was isolated with a chilled 18-gauge blunt needle, incubated in 50 μl of extraction buffer (Arcturus PicoPure RNA Isolation Kit; Applied Biosystems) at 42 °C for 30 min, briefly vortexed, and stored at −80 °C. RNA was isolated according to the Arcturus PicoPure kit manual, as previously reported. RNA purification columns were conditioned with 250 μl conditioning buffer for 5 min. Fifty microliters of 70% alcohol was added to each sample, mixed thoroughly, transferred to the conditioned column, and centrifuged to collect the RNA. Columns were washed with 100 μl Wash Buffer #1 and DNAse treated (5 μl DNAse I+35 μl RDD Buffer per sample). Columns were washed again with 40 μl Wash Buffer #1 and twice with 100 μl Wash Buffer #2. Each column was transferred to a new microcentrifuge tube and RNA was eluted using 15 μl elution buffer. Samples were frozen at −80 °C until RNA quality readings were obtained.

To determine RNA quality, samples were thawed, spectrophotometer readings were obtained, and samples meeting criterion (260/280 values between 1.80 and 2.20) were diluted to match the RNA concentration of the least concentrated sample. Samples were DNase-treated at 42 °C for five mins and then underwent first strand cDNA synthesis upon addition of the reverse transcriptase cocktail from the RT^2^ First Strand kit (primer and external control mix, reverse transcriptase enzyme mix, reverse transcriptase buffer, and H_2_O, in ratios of 1:2:4:3). Synthesized cDNA samples were diluted with a cocktail containing the RT^2^ SYBR Green Master Mix (Qiagen), and 25 μl of the mixture was deposited into each well of a custom-designed RT^2^ Profiler Array for analysis by a MX3000P real-time thermal cycler (Stratagene, San Diego, CA, USA).

A quantittive PCR (qPCR) approach was taken instead of microarray because a microarray would require amplification of the small amount of RNA collected from EWcp, and amplification may be subject to disproportional distortion of quantitative gene amounts. qPCR analyses were done as biological but not technical replicates, due to the high number of housekeeping genes and additional controls already included on each array. Only mice with EWcp samples that met high-quality RNA criteria (260/280 values between 1.80 and 2.20, concentrations⩾5.40 ng μl^−1^) were used for the gene expression and drinking analyses (*n*=9–13 per group).

The 48 wells analyzed for each animal included wells for: five RT and genomic DNA controls, six housekeeping genes (Supplementary Information; Supplementary Table S1), 23 EWcp-enriched genes of interest, three genes encoding inducible transcription factors (ITFs), eight dopamine-related genes, and three CRF-related genes other than *Ucn* (Supplementary Table S2). Mean cycle thresholds (CTs) for the six housekeeping genes included on the qPCR array were first normalized to 18S mRNA levels (diluted 1:100 000), and then compared between Alcohol and the matched Naive control group.

Following normalization to 18S, mRNA levels of housekeeping genes were similar between groups, excluding *Gusb* (*t*_20_=3.15; *p* <0.01). Thus, all genes of interest were normalized to the average of the remaining housekeeping genes: *Actb*, *Gapdh*, *Hprt*, *Hsp90ab1*, and *Reep5*. For each individual gene of interest, mean CT values were normalized by the equation 2^−ΔCT^, where ΔCT=the CT for the gene of interest subtracted from the mean CT value of the housekeeping genes, and compared between groups by t-test (adjusted significance threshold to account for multiple comparisons *P*<0.025). Normalized mRNA levels of 17/37 genes of interest (including *Ucn* and other neuropeptide-related transcripts) were significantly elevated in Alcohol mice, relative to H_2_O mice (Supplementary Table S3). Significance threshold was set at *p*<0.05 because we specifically hypothesized alcohol-induced upregulation of *Ucn, Fos*, and several other neuropeptide-related transcripts. In addition, the majority of genes assessed were already known to be specifically enriched within EWcp.^32^ Correlational analyses performed on the Alcohol group aimed to identify relationships between mRNA levels and measures of alcohol intake (significance threshold *P*<0.025). As controls, we also correlated the mean water and food intake from 20% alcohol days with the mRNA levels for genes that were significantly correlated with alcohol intake.

### Lentiviral targeting strategy

Lentiviruses were designed similar to others from the Chen group designed to target specific components of the CRF system.^42^ Lentiviruses contained a shRNA targeted against either the mouse *Ucn* sequence or a control sequence directed against the mouse *Ucn2* gene. shRNAs were tagged with GFP and expressed under the constitutive H1 promoter. The italic part is the *Ucn* sequence (375–393), the bold and underlined is the shRNA loop, and the rest is the sequence required for the cloning into the viral construct and the H1 promoter:

5′CTGTCTAGACAAAAA*ACCTCACCTTCCACCTGCT***TCTCTTGAA***AGCAGGTGGAAGGTGAGGT*GGGGATCTGTGGTCTATACA. The control lentivirus sequence is: 5′CTGTCTAGACAAAAA*TCAAATACTAGCCCATGTT***TCTCTTGAA***AACATGGGCTAGTATTTGA*GGGGATCTGTGGTCTCATACA. The *Ucn2*-shRNA was chosen as a control sequence because it is distinct from any sequence that is complementary to the *Ucn* gene, yet the remainder of the vector is identical between the two viruses. Using the *Ucn* and *Ucn2* viruses in the EWcp is a favorable approach because the EWcp contains only *Ucn* and not *Ucn2*.

### Surgical procedures

Anesthesia was induced with 5% isoflurane delivered in oxygen and maintained at 1–2%. Mice received 0.30 ml  subcutaneous Carprofen, and underwent stereotactic lentiviral infusion surgery. A small hole was drilled in the left skull at −3.50 mm (A/P) and +1.00 mm (M/L) from bregma. A borosillicate glass micropipette injector was lowered 4.30 mm into the brain at a 15° angle, terminating in the middle EW at a depth of 3.90 mm beneath the skull (D/V), along the midline. One microliter of the Ucn1 shRNA virus or control virus was infused via a 5.0 μl Hamilton syringe connected to the injector via plastic tubing over the course of 5 min, and the injector remained in place for an additional 5 min following infusion. Mice initially recovered for 1 h in a fresh warm cage placed on a heating pad.

### Effects of EWcp-Ucn1 knockdown on long-term intermittent alcohol drinking

Mice received lentiviral surgery (*n*=15–20 per viral group), and following the alcohol-free baseline, underwent the long-term intermittent drinking procedure described previously, with *n*=2 per viral group serving as alcohol-naive water-only controls. For analysis of behavioral data, RM-ANOVA was performed across all 20% alcohol days (between-subjects factor of virus, with day as the repeated measure).

### Immunohistochemical analyses

Analyses were performed similarly to previously described studies from our lab.^40^ Brains were dissected and stored in 2% PFA in PBS overnight. Brains were then transferred to 20% sucrose in PBS for 24 h prior to being stored in 30% sucrose in PBS for 24–96 h prior to being sliced in 30 μm sections on a Leica cryostat (Wetzlar, Germany). Sections from the EWcp were collected in 0.10% NaN_3_ in PBS for later immunohistochemical staining and verification of adequate lentiviral infection. Midbrain slices were stained for Ucn1 using the rabbit anti-Ucn1 (1:5000, #SAB4300830, Sigma, St. Louis, MO, USA) and underwent secondary labeling with diaminobenzidine (#34002, ThermoFisher, Waltham, MA, USA) or Alexa-594 (1:500, #A-11012, ThermoFisher).

Only subjects with extensive GFP labeling within the EWcp were included in KD analyses. For KD analysis at 3 weeks post-surgery, the number of DAB-stained Ucn1-positive cells in the EWcp were counted (4–6 EWcp sections per subject, *n*=2 per group). For analysis of KD at 8 weeks post-surgery, we obtained × 20 photomicrographs of EWcp-Ucn1 immunofluorescence (4-6 EWcp sections per subject, *n*=7–10 per group), and used ImageJ (National Institutes of Health) to determine the density of Ucn1-IR. Brightness and exposure settings were matched for all slices across all subjects, and data were analyzed under blinded conditions. Measures of EWcp-Ucn1 KD were analyzed by RM-ANOVA (between-subjects factor of virus, with EWcp bregma level as the repeated measure). Anterior, middle, and posterior EWcp were defined as −3.30 to −3.50, −3.50 to −3.70, and −3.70 to −3.90 mm from bregma, respectively.

## Results

**Figure 1:**
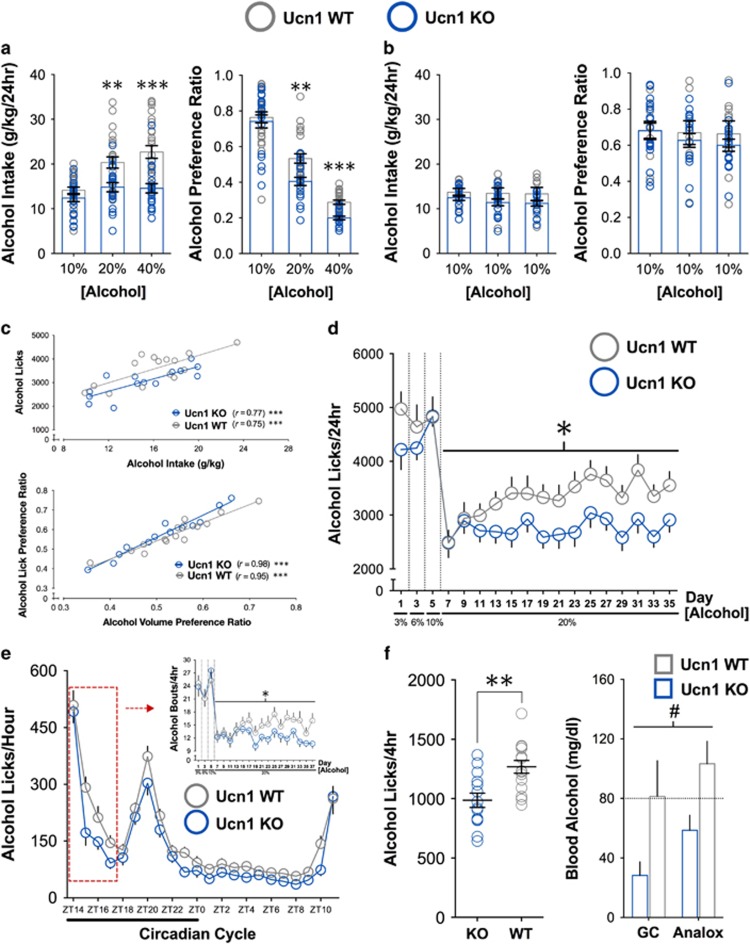
Ucn1 Deletion selectively reduces the progressive escalation of long-term voluntary binge alcohol consumption. (**a**) Deletion of Ucn1 reduced alcohol intake (left) and preference (right) in dose-escalated continuous access drinking (intake: genotype × concentration interaction F_2,84_=10.12, *P*=0.0001, preference: genotype × concentration interaction F_2,84_=2.88, *P*=0.06; Bonferroni *post hoc* **P<0.005 and ***P<0.0005 vs WT). (**b**) Deletion of Ucn1 did not alter alcohol intake (left) or preference (right) in non-escalated continuous access drinking. (**c**) Alcohol licks (Top) and alcohol lick preference ratios (Bottom) were each significantly positively correlated with measurements of alcohol intake and preference in both genotypes (all ***P<0.002, *r*^2^>0.56). (**d**) Across 20% alcohol days, deletion of Ucn1 blunted the progressively escalating pattern of alcohol licks observed in WT controls (linear regression F_1,26_=7.63, *P=0.01). (**e**) Hourly analysis of the 20% alcohol lick timecourse identified the initial 4 h containing the largest magnitude of genotype differences. Across these 4 h periods in the 20% alcohol phase (Inset), deletion of Ucn1 blunted the progressive escalation of alcohol-drinking bouts observed in WT controls (linear regression F_1,28_=8.25, *P<0.01). (**f**) Relative to WT mice, Ucn1 KO mice had significantly fewer alcohol licks during the final 4 h session (left) (*t*_27_=3.50, **P<0.005), resulting in BACs that were significantly lower than those of WT mice and below the binge threshold (right) (#main effect of genotype F_1,27_=5.40, *P*<0.05). In both genotypes, BAC values obtained by each method were highly positively correlated (both *r*>0.94, *p*<0.0001). GC, gas chromatography; KO, knockout; WT, wild-type; ZT, zeitgeiber time (ZT 0=lights-on).

To investigate a modulatory role for Ucn1, we tested male and female Ucn1 KO mice and WT littermates. Genetic deletion of Ucn1 reduced high levels of intake and preference across 2 weeks of continuous access drinking when alcohol concentrations were increased from 10 to 20 to 40% (Figure 1a). However, Ucn1 deletion did not alter low-level drinking when the alcohol concentration remained at 10% (Figure 1b), showing that progressive escalation of intake is an important characteristic of Ucn1's contribution to free-choice drinking. To precisely evaluate Ucn1's role in the escalation phenotype, we used lickometer devices^43^ to track the drinking microstructure across 5 weeks of intermittent access, in which mice were offered subsequently increasing concentrations of alcohol during every-other-day drinking sessions. Alcohol bottle licks and lick preference ratios were positively correlated with standard measures of alcohol intake and preference in both genotypes (Figure 1c). Across days, WT mice progressively increased licks for 20% alcohol. This pattern was significantly blunted in Ucn1 KO mice, indicating that Ucn1 is necessary for patterns of excessive drinking reminiscent of the transition to addiction (Figure 1d). Hour-by-hour analysis of circadian drinking patterns highlighted a 4 h window in the dark cycle during which Ucn1 deletion reduced bouts of 20% alcohol licks (Figure 1e). Replicate analysis of blood samples via two independent methods confirmed that Ucn1 KO mice failed to reach the binge BAC levels observed in WT controls following a final 4 h drinking session (Figure 1f). Data separated by sex are shown in Supplementary Figures S1 and S2.

Separate tests concluded that Ucn1 deletion did not alter sweet and bitter taste reactivity (Supplementary Figure S3), alcohol-induced loss-of-righting-reflex (Supplementary Figure S4), baseline anxiety-like behavior (Supplementary Figure S5 and S6), alcohol-induced hypothermia (Supplementary Figure S7), nor delay discounting measures of impulsive-like behavior (Supplementary Figure S8). Analyses of short-term limited-access alcohol preference and alcohol-induced locomotor suppression identified marginal sex-specific genotype effects (Supplementary Figure S9 and S10), but the absence of systematic sex effects supported the use of males for further studies on the association of Ucn1 with escalated drinking.

**Figure 2:**
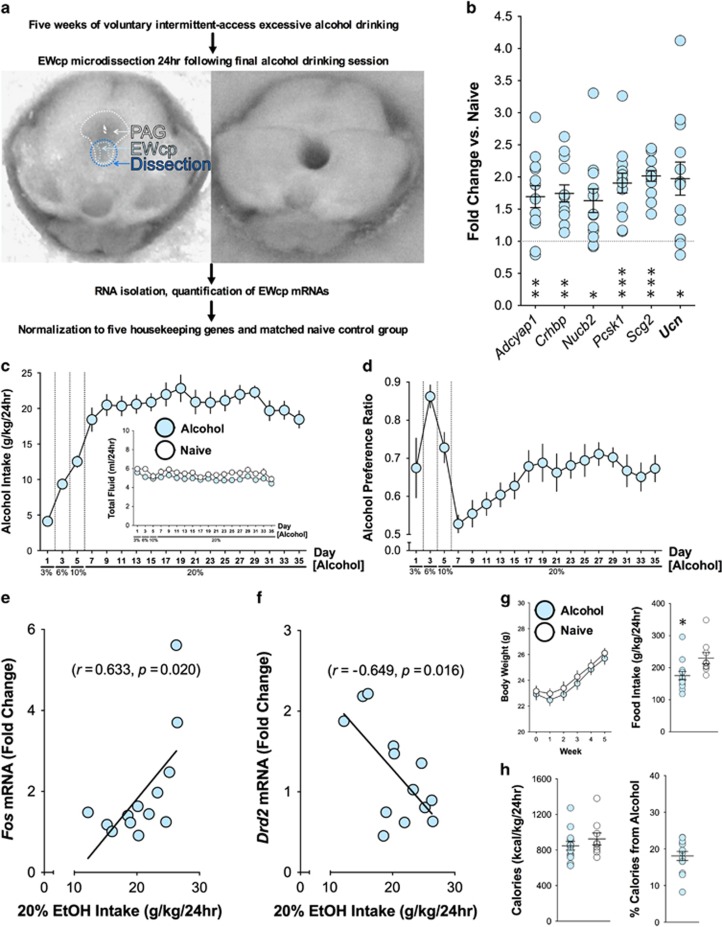
Long-term intermittent excessive alcohol consumption activates neuropeptide-related gene expression in the EWcp**.** (**a**) Experimental summary and schematic of midbrain slice EWcp microdissection. (**b**) 24 h following the end of the final alcohol-drinking session, a subset of neuropeptide-related genes were significantly upregulated in the EWcp of Alcohol mice relative to water controls (all *t*_20_⩾2.57, *P<0.025, **P<0.005, ***P<0.0005) (**c**) During the 20% alcohol phase, intakes stabilized at high levels of ~21 g kg^−1^ per 24 h. Total fluid intakes (Inset) were nearly identical between groups. (**d**) Alcohol preference escalated progressively across 20% days, reaching significantly higher levels on Days 15 through 35, relative to Day 7 (RM-ANOVA F_14,168_=8.21, *P*<0.0001; Bonferroni post hoc comparisons *P*⩽0.0007). (**e**) Daily 20% alcohol intake was significantly positively correlated with EWcp *Fos* mRNA levels and (**f**) significantly negatively correlated with *Drd2* mRNA levels (both *r*^2^>0.40, *P*<0.025). (**g**) Groups did not differ in body weights, and daily food intake on 20% alcohol-drinking days was slightly decreased in alcohol-drinking mice relative to controls (*t_20_=2.60, *P*<0.05). (**h**) Total daily caloric intake was similar between groups, as alcohol-drinking mice received an average of 18% of their total calories from alcohol. Midbrain dissection schematic in **a** re-used with permission.^32^ EWcp, Edinger-Westphal nucleus.

Given the sensitivity of EWcp-Ucn1 neurons to alcohol^22^ and present evidence supporting a specific role for Ucn1 in escalating alcohol drinking, we hypothesized that repeated binge drinking sessions would increase gene expression of Ucn1 and other neuropeptide transcripts within the EWcp. We designed custom arrays based on our prior characterization of the EWcp expression profile^32^ (Supplementary Tables S1 and S2), and microdissected EWcp samples from intermittent alcohol-drinking mice and matched water-drinking controls for subsequent quantification of gene expression (Figure 2a). Across 5 weeks of every-other-day drinking, mice achieved stable intakes of ~21 g kg^−1^ per 24 h that were accompanied by a progressive escalation in preference for 20% alcohol (Figures 2c and d). Quantification of housekeeping-normalized mRNA at 24 h post-alcohol identified six neuropeptide-related transcripts (including *Ucn*) that were significantly elevated in alcohol-drinking mice, relative to controls (Figure 2b, Supplementary Table S3). Mean daily 20% alcohol intake was correlated (positively) with levels of *Fos* mRNA and (negatively) with levels of *Drd2* mRNA (Figures 2e and f). Neither transcript was significantly correlated with food or water intake. Body weights and caloric intakes were nearly identical between groups, as alcohol-drinking mice received ~20% of their daily calories from alcohol itself (Figures 2g and h).

**Figure 3:**
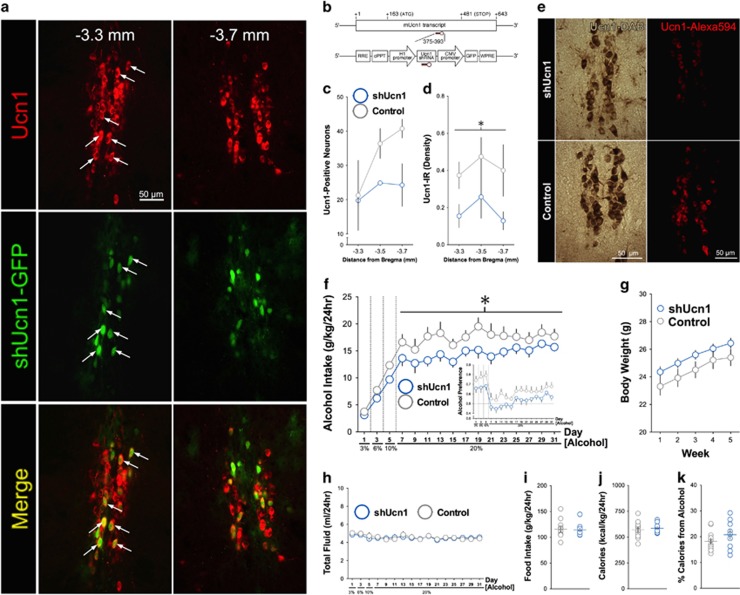
Ucn1 knockdown in the EWcp selectively reduces long-term excessive alcohol drinking. (**a**) shUcn1-GFP lentivirus was expressed primarily in Ucn1-positive neurons at multiple bregma levels throughout the rostral-caudal axis of EWcp, indicated by co-localization of GFP in neurons immunostained for Ucn1 (white arrows). (**b**) Lentiviral vector driving GFP-tagged Ucn1 shRNA sequence. (**c**) Three weeks following surgery, number of Ucn1-positive neurons was reduced throughout the medial and posterior subregions of EWcp. (**d**) Eight weeks following surgery, density of Ucn1 immunoreactivity was significantly lower overall in the EWcp of shUcn1 vs Control mice (main effect of virus; F_1,30_=4.99, *P<0.05). (**e**) Images demonstrating that shUcn1 virus reduced the number of Ucn1-positive neurons as assessed by diaminobenzidine (DAB) staining (left) and the density of Ucn1 immunoreactivity as assessed by immunfluorescence staining (right). (**f**) EWcp-shUcn1 KD significantly reduced alcohol intake across the long-term 20% alcohol drinking phase (main effect of virus; F_1,228_=4.52, *P<0.05), and reduction in alcohol preference was strongly trending toward significance (*P*=0.055, Inset). Groups did not differ in (**g**) body weight, (**h**) total fluid consumption, (**i**) average food intake, (**j**) caloric intake nor (**k**) percent calories consumed from alcohol. EWcp, Edinger-Westphal nucleus; GFP, green florescent protein.

After confirming Ucn1 regulation by escalated alcohol consumption, we set out to investigate a causal role for Ucn1 within the EWcp and eliminate the possibility that Ucn1 KO effects were due to developmental compensations. We performed EWcp-targeted RNA interference-mediated knockdown using a lentivirus encoding a GFP-tagged short hairpin RNA directed against Ucn1 (shUcn1; Figure 3b). Lentiviral shUcn1-GFP expression was localized to Ucn1-positive neurons throughout the EWcp (Figure 3a). Relative to viral controls, Ucn1 cells and Ucn1 immunoreactivity were decreased in the EWcp of shUcn1 mice several weeks following surgery (Figures 3c–e). Consilient with the global Ucn1 KO phenotype, shUcn1 lentiviral knockdown significantly reduced long-term intermittent access alcohol intake (Figure 3f), doing so in the absence of changes to body weight, total fluid intake, food intake, and total caloric consumption (Figures 3g–k).

## Discussion

In summary, we documented alcohol-induced fluctuations in neuropeptide gene expression within these stress-responsive midbrain neurons, and provided converging lines of evidence that EWcp-Ucn1 activity is essential for chronic escalated alcohol drinking. Together with previous observations, these studies point to contrasting contributions of CRF system ligands to the development of excessive alcohol use. For example, the current data indicate that Ucn1 regulates escalation of drinking via alcohol-specific mechanisms, whereas CRF contributes to non-escalating binge alcohol drinking^44^ through regulation of overall food and fluid intake.^38^ On the other hand, substantial amount of research shows that CRF is critical for established excessive alcohol intake under conditions of drug dependence and stress-enhanced drinking.^45^

The EWcp profiling experiment highlighted an active role for Ucn1 that complemented knockout and knockdown studies, and provided a readout of additional high-quality candidates to interrogate in future studies. Alcohol-induced upregulation of *Adcyap1* (PACAP) and *Nucb2* (nesfatin-1) pointed toward two previously overlooked neuropeptide candidates that may also mediate alcohol drinking via release from EWcp neurons. Yet, our findings specifically implicate Ucn1 in the progressive escalation of alcohol intake. Upregulated levels of *Pcsk1* (proprotein convertase subtilisin/kexin type 1) and *Scg2* (secretogranin II) in alcohol-experienced mice further support our hypothesis that alcohol-induced changes in EWcp function involve elevated rates of neuropeptide enzymatic processing and packaging for release, respectively.^46, 47^ Given the dose-dependent activation of EWcp-cFos protein expression in response to alcohol,^22^ the positive correlation between alcohol intake and EWcp-*Fos* mRNA was strongly expected, emphasizing the reliability of our approach to detect alcohol-induced changes in EWcp candidate genes. Furthermore, the negative relationship between *Drd2* mRNA and alcohol intake suggests that long-term alcohol drinking is associated with a loss of dopamine D2 receptor-mediated inhibition in EWcp neurons (or dopaminergic neurons of the rostral linear nucleus of the raphe, which are intermingled throughout the EWcp field).^48^

All EWcp-enriched neuropeptides have documented anxiogenic functions, so their enhanced activities may reflect a heightened stress-like state that occurs following cessation of long-term drinking. Similar to amygdala and ventral tegmental CRF system adaptations observed during drug dependence,^3, 49^ we hypothesize that EWcp-Ucn1 neurons are susceptible to allostatic processes that perpetuate the addiction cycle via persistent changes following repeated cycles of alcohol intoxication and withdrawal. Recent investigations of CRF system contributions to binge alcohol drinking focused on the ventral tegmental area and dorsal raphe nucleus.^14, 16^ These regions (as well as the lateral septum) are likely relevant to the outcomes in the current study, as our group demonstrated functional EWcp-Ucn1 interactions with these structures,^24, 30, 50, 51^ hinting at novel midbrain circuits linking the stress response to dysregulated drug-seeking.^22, 23, 52^

Finally, our observation of upregulated CRF binding protein gene expression in the EWcp of alcohol-drinking mice suggests that continued study of CRF system pharmacology within the context of addiction neurocircuitry will clarify EWcp-Ucn1's role in motivation for alcohol. Importantly, clinical studies in alcoholic populations that targeted only the type-1 CRF receptor produced undesirable results.^53, 54^ Because Ucn1 acts with high affinity at both CRF receptors, as well as CRF binding protein, our findings should lead the way toward novel multi-pronged therapeutic strategies for mitigating chronic alcohol abuse.
