# Supplementary material for: Control of chronic excessive alcohol drinking by genetic manipulation of the Edinger–Westphal nucleus urocortin-1 neuropeptide system
Source: Transl Psychiatry. 2017 Jan 31;7(1):e1021–. doi: 10.1038/tp.2016.293 (PMC5299395; doi:10.1038/tp.2016.293)
Supplement: Supplementary Methods [file tp2016293x1.docx]

**SUPPLEMENTAL METHODS**

***Figures S1 and S2: Effects of Ucn1 KO on Alcohol Drinking in Female and Male Mice***

Methods described in main text.

***Figure S3: Effects of Ucn1 KO on Two-Bottle Choice Taste Reactivity***

Male and female Ucn1 KO and WT mice were used (n = 7-11 per sex, per genotype). On the first day following habituation to the reverse light-dark cycle (Day 1) at two hours into the dark cycle, H2O in one of the two 25 ml bottles was replaced with 3% sucrose and remained on the cage top for four consecutive days. On Days 5-8, the concentration of sucrose was increased to 10%. On Days 9-12 and 13-16, sucrose was replaced with .003% and .015% saccharin, respectively. On Days 17-20 and 21-24, saccharin was replaced with .03 and .06 millimolar quinine, respectively. Bottles were read daily at two hours into the dark cycle, and food was weighed on the final two days of each tastant concentration phase. Initial statistical analyses did not identify any interactions with tastant concentrations, thus concentrations were combined for analyses. Sex x genotype interactions for tastant intake, tastant preference, and total fluid intake failed to reach statistical significance (*F*_1,22_ = 0.53, 0.26, 0.46; *p* = .47, .61, .50), thus sexes were combined for all analyses. Tastant intake and preference, food intake, and total caloric intake were analyzed by two-way RM-ANOVA (genotype as the between-subjects factor, tastant as the repeated measure).

***Figure S4: Effects of Ucn1 KO on Alcohol-Induced Sedation***

Following tastant drinking, mice from the experiment resumed normal H2O drinking and were undisturbed for four days before being tested for their sensitivity to Alcohol-induced sedation using the loss of righting reflex (LORR) procedure on three consecutive days. At four hours into the dark cycle, mice were injected i.p. with a 4.0 g/kg dose of 20% Alcohol (v/v) dissolved in saline (injection volume of 25.35 ml/kg), and placed in a holding cage until they appeared to be intoxicated. Mice were then placed on their backs in a V-shaped trough, and time of LORR began when mice were no longer able to right themselves within 30 seconds of being placed on their backs. Mice were observed for the following 2-3 hours. When mice righted themselves, they were repeatedly placed on their backs again until they were able to right themselves twice within 30 seconds.

The time was recorded at the moment of injection, the moment of LORR, and the moment of re-gain of righting reflex on each of the three days. On the third day, immediately following the re-gain of righting reflex, mice were euthanized by CO2 inhalation, and trunk blood samples were obtained for later BAC analysis. Sex x genotype interactions for LORR latency and LORR duration failed to reach statistical significance (*F*_1,27_ = 0.36, 0.11; *p* = .56, .75), thus sexes were combined for all analyses. LORR latency and duration were analyzed separately by two-way RM-ANOVA (between-subjects factor of genotype, with day as the repeated measure). BACs on Day 3 were compared between genotypes by t-test.

***Figure S5, S6: Effects of Ucn1 KO on Baseline Anxiety-Like Behavior***

Male and female Ucn1 KO and WT mice (n = 10-15 per sex, per genotype) were tested in two widely-used rodent models of anxiety-like behavior: the elevated plus maze (EPM) and the light-dark box (LDB). Group-housed mice were moved to a dark experimental room and allowed to habituate for 1hr prior to testing on each day. Testing occurred between two and eight hours into the light cycle on several consecutive days in which each mouse underwent both the EPM and the LDB in a fully counterbalanced order, with one day of rest between each test, in order to minimize the potential effects of repeated testing on subsequent behavior.

The EPM apparatus (Med Associates, Inc., St. Albans, VT, USA) consisted of two black opaque high-walled arms and two white open arms (51-cm long x 8-cm wide) elevated 60 cm off the ground. Small lamps were placed over the open arms, and the closed arms remained un-lit, resulting in respective lux values of 95 and 2. Mice were placed in the center platform facing a closed arm, and the following variables were scored live during a 5 min test: latency to enter open arm, entries and time spent in open and closed arms, number of head dips over the sides of open arms, rearing behavior, grooming, urination, and fecal boli. Between each session, the EPM was cleaned with H2O and a sponge, and thoroughly dried with paper towels.

The LDB (black/white box, John Crabbe Laboratory) consisted of a two-chambered apparatus comprised of one white acrylic box (28 x 28 x 30 cm) connected to a smaller black acrylic box (28 x 17 x 30 cm). A small doorway in the shared wall of the boxes allows movement between the two compartments. A small lamp was placed over the light side, and the dark side remained unlit, resulting in respective lux values of 195 and 2. Mice were placed in the dark side of the apparatus facing a dark corner, and the following variables were scored live during a 5 min test: latency to enter light side, number of dark-light and light-dark transitions, time spent in light side, rearing behavior, grooming, urination, and fecal boli. Between each session, the LDB was cleaned with H2O and a sponge, and thoroughly dried with paper towels.

Initial analyses uncovered no significant interactions with factors of order (EPM/LDB vs. LDB/EPM), thus order groups were combined for final analyses. Sex x genotype interactions for all EPM and LDB variables failed to reach statistical significance (*F*_1,42_ = 0.01-3.92; *p* = .938-.054), thus sexes were combined for all analyses. Genotypes were compared on the scored measures by t-test.

***Figure S7: Effects of Ucn1 KO on Alcohol-Induced Hypothermia***

Male and female Ucn1 KO and WT mice (n=10-15 per sex, per genotype) were injected i.p. with saline or 2 g/kg alcohol during two separate tests, and core body temperatures were recorded by rectal thermometer readings at 0, 15, 30, 60, 90, and 120 minutes following injection. Timecourse analysis of alcohol-induced hypothermia did not identify significant sex x genotype interaction, so sexes were combined for analyses. RM-ANOVA (repeated measure of Time) did not identify any significant genotype effects.

***Figure S8: Effects of Ucn1 KO on Impulsive-Like Behavior***

Male and female Ucn1 KO and WT mice participated in two cohorts (n=8-17 per sex, per genotype). Mice completed the experiment in two squad, and began operant training at 15-25 weeks of age. The first training day followed a minimum of 48 hours on a food-restricted diet (approximately 2 g of mouse chow/day/mouse), and mice were maintained at approximately 90% of age-adjusted free-feeding weight throughout to ensure they would be motivated to respond for sucrose.

Behavioral testing occurred in operant chambers housed in sound-attenuating ventilated boxes (Med-Associates, St. Albans, VT). In all chambers, a 100 mA house light and a clicker to signal reward delivery were mounted on the outside of the back panel. One wall panel contained three nosepoke holes mounted 1.27 cm above the grid floor. Each contained a liquid cup and sensors to identify when nosepokes occurred. Immediately above each cup was a yellow LED light. Computer-controlled pumps were used to deliver 10% w/v sucrose to the liquid cups. All input and output was controlled and recorded using programs written in MED-PC (Med-Associates Inc.).

Delay discounting behavior was measured using the adjusting amount procedure (Mitchell, 2014). Each session lasted until animals either completed 60 free-choice trials or 60 min elapsed. Each trial began with illumination of the house light and center LED cue light. To control for right- or left-side preference, mice had to complete a center nose poke, after which the center LED shut off and the left and right nose poke LED cue lights turned on. A nose poke to the “immediate reward” side turned off the cue lights and sucrose was delivered; if the “delayed reward” side was chosen, the cue light on the immediate side extinguished but the LED on the delayed side remained on for the duration of the delay.

The initial amount of sucrose delivered for choice of the immediate side was 10 µl. This amount increased by 10% each time the mouse chose the delayed side and decreased by 10% when the subject chose the immediate alternative. The reward delivered for choices of the delayed side was fixed at 20 µl. An external sound-generator produced a 10-Hz click when sucrose was delivered. If a subject selected the same side on two consecutive trials, the next trial was a forced choice trial on the other side.

Mice experienced 6 training phases (Mitchell, 2014). After Phase 6 training was complete, either the left or right nose poke was designated as the “delayed reward side” and the other nose poke as the “immediate reward side” (counterbalanced between subjects). Then, on successive sessions, the delays prior to food delivery from the delayed reward side were increased to familiarize subjects with the delays (1^st^ session: 0 s, 2^nd^ session: 1 s, then 2, 4, and 8 s). Subjects then began the experimental phase.

During the experimental phase, the 5 delays were implemented on different sessions according to a pseudo-Latin Square design. Mice experienced the adjusting amount procedure until they completed at least 10 sessions for all 5 delays and data from all sessions were used as long as more than 45of the 60 trials were completed (mean for each delay > 8 sessions).

The main dependent variable from the delay discounting task was the median amount of sucrose solution available from the immediate alternative calculated from trial 31until the final trial of the session. Other dependent variables included reaction time (RT), was defined as the time, in seconds, between the initial center cue light turning on and the center nose poke. Reaction times were further delineated by whether they were followed by a nose poke on the delayed reward side (RT1) or the immediate adjusting side (RT2). Also choice reaction time (CHRT) was examined, defined as the time, in seconds, between the center nose poke and the choice nose poke, either delayed (CHRT1) or immediate (CHRT2).

Analyses were conducted using mixed factor analyses of variance (ANOVAs) with line and sex as the between-subjects factors and delay as the within-subjects factor. ANOVAs with an additional within subjects factor (choice: immediate or delayed side) were conducted on reaction time, and choice reaction time data. Within subject analyses were corrected for violations in sphericity using the Huynh-Feldt correction, and these corrected degrees of freedom are shown where appropriate.

As expected, increases in delay to reward resulted in significantly lower indifference points (*F*(2.9, 135.2) = 58.89, *p* < .001). This decrease in indifference points, however, was not different for the KO than the WT mice (*p* = 0.54). As observed in other studies (Helms et al. 2006; Wilhelm & Mitchell 2009, 2012), after a trial began, as signaled by illuminating the light over the center nosepoke hole, the latency with which mice nosepoked to initiate the trials was systematically longer as the delay to the delayed reward increased (*F*(3.2, 172.3) = 71.80, *p* < .001) regardless of line or sex, or whether the delayed or immediate reward side was subsequently selected (no main effecs nor interaction: all *p*s > .10). A similar pattern emerged for choice reaction times, in that mice of both lines responded more slowly on sessions where the delay to the delayed reward was longer (*F*(2.9, 140.7) = 13.33, *p* < .001) with no main effects nor interactions with line, sex or chosen side.

***Figure S9: Effects of Ucn1 KO on Two-Bottle Choice Drinking-in-the-Dark***

Male and female Ucn1 KO and WT mice (n= 3-12 per sex, per genotype) were used. For the first three days following acclimation to the reverse dark-light cycle (Days 1-3), two hours into the dark cycle, the two 25 ml H2O bottles on each cage top were replaced with two small 10 ml plastic bottles (for more precise measurement of volumes), one containing H2O and one containing 15% alcohol. Four hours into the dark cycle, the 10 ml bottles were removed and replaced with the original 25 ml H2O bottles. With each exchange of 25 ml and 10 ml bottles, volumes were recorded to the nearest 0.1 ml. On Day 4, the Alcohol drinking session was extended from 2hr to 4hr, and food was collected and weighed from the cage tops at the beginning and end of the session. During food weighing, the cage floor was checked for pieces of food pellets that may have fallen through the cage top, and if found, were included in food weight measurements. Following the end of the 4hr session on Day 4, mice were euthanized by carbon dioxide (CO2) inhalation, and trunk blood was collected for later analysis of blood alcohol concentrations (BACs) by the Analox method, as previously reported[^1^](#_ENREF_1). Alcohol intake (g/kg), Alcohol preference, food intake (g/kg), total caloric intake from Alcohol and food combined (kilocalories [kcal] per kg), and percent calories consumed from Alcohol were the dependent variables. Days 1-3 data were analyzed by three-way RM-ANOVA (between-subjects factors of sex and genotype, with day as the repeated measure). Day 4 data were analyzed by two-way ANOVA (between-subjects factors of sex and genotype). Significant interactions with genotype were followed up with simple main effect comparisons individually at each level of sex.

***Figure S10: Effects of Ucn1 KO on Alcohol-Induced Locomotor Activity***

Male and female Ucn1 KO and WT mice (n = 7-9 per sex, per genotype) were tested for sensitivity to Alcohol-induced locomotor activity. Group-housed mice were moved to the experimental room and given 1hr to habituate before receiving an i.p. injection and undergoing a 15-min locomotor activity test in one of four sound-attenuated behavioral chambers. Horizontal locomotor activity was detected by interruption of a 10 x 12 array of photocell beams equally spaced at a height of 1 cm along the walls of a 21 x 25 x 18 cm enclosure with a steel bar grid floor (San Diego Instruments; San Diego, CA). This chamber resided within a larger sound-attenuating box containing a fan and houselight. Horizontal activity was defined as the total number of photocell beam breaks during the 15-minute tests. Testing occurred between four and eight hours into the light cycle. On Days 1 and 2, mice received 17 ml/kg saline (i.p.) and activity levels were measured immediately afterward to allow habituation to the apparatus (Day 1), and to measure baseline activity levels (Day 2). On Days 3, 5, 7, 9, and 11, mice received doses of 0.00, 0.75, 1.50, 2.00, and 2.75 g/kg Alcohol dissolved in saline, administered in concentrations of 0.0%, 5.6%, 11.2%, 14.9%, and 20.5% (v/v), always with an injection volume of 17 ml/kg. The injection volume rather than the concentration of Alcohol remained constant in order to match the volume injected on the first two saline habituation days. The order of Alcohol doses was fully counterbalanced, and all mice received a day of rest between each Alcohol testing session to minimize potential impact of pharmacological tolerance on the locomotor effects of Alcohol.

The number of horizontal beam breaks during each 15 min session was used as the dependent variable, and data were analyzed by RM-ANOVA (between-subjects factors of genotype and sex). To assess potential differences in habituation and baseline activity, Days 1 and 2 were analyzed with day as the repeated measure. To assess effects of Alcohol on locomotor activity, Days 3, 5, 7, 9, and 11 data were analyzed with dose as the repeated measure (0.00, 0.75, 1.50, 2.00, 2.75). Significant interactions were followed up with Bonferroni-corrected post-hoc comparisons.

**SUPPLEMENTAL FIGURE LEGENDS**

**Figure S1: Effects of Ucn1 KO on Escalated and Non-Escalated Continuous Access Alcohol Drinking in Female vs. Male Mice**

**(Top):** Female mice. **(Bottom):** Male mice. **(a, c)** Deletion of Ucn1 reduced alcohol intake and preference in the escalated procedure. Intake and preference analyses failed to identify significant sex x genotype interactions (*F*_1,40_ = 3.82, 0.12; *p* = 0.06, 0.73) **(b, d)** Deletion of Ucn1 had no significant effect on intake and preference in the non-escalated procedure. Intake and preference analyses failed to identify significant sex x genotype interactions (*F*_1,22_ = 0.01, 0.32; *p* = 0.92, 0.58).

**Figure S2: Effects of Ucn1 KO on Escalated Intermittent Access Alcohol Drinking in Female vs. Male Mice**

**(Left):** Female mice. **(Right):** Male mice. **(a, b)** Deletion of Ucn1 reduced daily alcohol licks across the intermittent access drinking study. Analysis failed to identify a significant sex x genotype interaction (*F*_1,25_ = 0.00; *p* = 0.96). **(c, d)** Deletion of Ucn1 reduced blood alcohol levels measured by Analox. Analysis failed to identify a significant sex x genotype interaction (*F*_1,25_ = 1.81; *p* = 0.19).

**Figure S3: Effects of Ucn1 KO on Continuous Access Tastant Drinking**

Ucn1 KO and WT mice did not differ in **(a)** tastant intake, **(b)** tastant preference, nor consumption of **(c)** food or **(d)** total calories during tastant access. Data are shown collapsed on sex and tastant concentration.

**Figure S4: Effects of Ucn1 KO on Alcohol-Induced Sedation**

Across three consecutive days of repeated testing with 4.0 g/kg Alcohol, deletion of Ucn1 had no effect on **(a)** the latency to loss-of-righting (LORR) onset, nor **(b)** the total LORR duration. **(c)** Blood alcohol concentrations (BACs) at time of regaining LORR on Day 3 were also similar between genotypes. Data are shown collapsed on sex.

**Figure S5: Effects of Ucn1 KO on Anxiety-Like Behavior (Elevated Plus Maze)**

Ucn1 KO and WT mice did not differ in **(a)** latency to enter an open arm, **(b)** time spent in the open arms, **(c)** open arm entries, **(d)** percent of arm entries into the open arm, **(e)** closed arm entries, **(f)** number of head dips over the side of open arms, or **(g)** number of grooming bouts. Panel **(h)** shows that relative to WT mice, Ucn1 KO mice displayed greater rearing behavior (**p* <.05). Data are shown collapsed on sex.

**Figure S6: Effects of Ucn1 KO on Anxiety-Like Behavior (Light-Dark Box)**

Ucn1 KO and WT mice did not differ in **(a)** latency to enter the light side of the box, **(b)** time spent in the light side of the box, **(c)** number of entries into the light side of the box, **(d)** total light/dark transitions, or **(e)** number of grooming bouts. Panel **(f)** shows that relative to WT mice, Ucn1 KO mice displayed greater rearing behavior (**p* <.05). Data are shown collapsed on sex.

**Figure S7: Effects of Ucn1 KO on Alcohol-Induced Hypothermia**

Ucn1 KO and WT mice did not differ in the timecourse of two separate tests (Left column, Right column) of core body temperature reactions to intraperitoneal injections of Saline (Top row), nor 2g/kg Alcohol (Bottom row). Data are shown collapsed on sex.

**Figure S8: Effects of Ucn1 KO on Delay Discounting**

Ucn1 KO and WT mice did not differ in impulsive-like behavior as measured by the variable-delay discounting procedure. Both Ucn1 KO and WT mice systematically chose a delayed 20 ul sucrose solution reward less as the delay to its delivery increased from 0 to 8 seconds, indicating that the value of this reward was discounted as a function of the delay to its receipt (*F*_4, 188_ = 58.89, *p* < .001). However, there were no strain nor sex differences nor interactions in the degree of discounting nor in any of the response latency measures (all *p*s > .05).

**Figure S9: Effects of Ucn1 KO on Two-Bottle Choice Drinking-in-the-Dark**

Data are from Day 4 of the study (no significant effects on Days 1-3). **(a)** Female mice weighed less than male mice, **(b)** consumed more total fluid per body weight than male mice, and **(c)** consumed more Alcohol per body weight than male mice overall. **(d)** Genetic deletion of Ucn1 decreased Alcohol preference only in male, but not in female mice. **(e)** Blood alcohol concentrations (BACs) did not differ between sexes and genotypes. **(f)** Female mice consumed more food than male mice overall. **(g)** Sexes and genotypes did not differ in total calories consumed or **(h)** percent calories consumed from Alcohol. Pound signs indicate significant main effects of sex (#*p* < .05, ##*p* < .005). Asterisk indicates significant simple main effect of genotype in the presence of a significant sex x genotype interaction (**p* < .05).

**Figure S10: Effects of Ucn1 KO on Alcohol-Induced Locomotor Activity**

Deletion of Ucn1 accentuated the locomotor depressant effects of 2.75 g/kg Alcohol in (Left) female mice, but not in (Right) male mice. Asterisks indicate significant sex x genotype x dose interaction and significant Bonferroni post-hoc comparison between female genotypes at 2.75 g/kg (****p* <.001).

**SUPPLEMENTARY TABLES**

**Table S1: List of Housekeeping Genes**

Asterisks indicate genes that were included in the average of housekeeping genes used to normalize the mRNA levels of the genes of interest.

| **Gene Symbol** | **Gene Name** |
| --- | --- |
| *18S* | 18S ribosomal RNA |
| *Actb** | Beta-actin |
| *Gapdh** | Glyceraldehyde 3-phosphate dehydrogenase |
| *Gusb* | Beta-glucuronidase |
| *Hprt** | Hypoxanthine-guanine phosphoribosyltransferase |
| *Hsp90ab1** | Heat shock protein 90-beta |
| *Reep5** | Receptor expression-enhancing protein 5 |

**Table S2. List of Genes of Interest**

Including six housekeeps and five controls, each half-array included 23 EW-enriched, 3 inducible transcription factor (ITF), 8 dopamine (DA)-related, and 3 CRF-related genes.

| **Gene Symbol** | **Category** | **Gene Name** |
| --- | --- | --- |
| *Adcyap1* | EW-Enriched | Pituitary adenylate cyclase-activating polypeptide |
| *Cart* | EW-Enriched | Cocaine- and amphetamine-regulated transcript |
| *Cck* | EW-Enriched | Cholecystokinin |
| *Cds2* | EW-Enriched | Phosphatidate cytidylyltransferase 2 |
| *Dlk1* | EW-Enriched | Delta-like homolog 1 |
| *Gabre* | EW-Enriched | GABA-A receptor subunit epsilon |
| *Gabrq* | EW-Enriched | GABA-A receptor subunit theta |
| *Ghsr* | EW-Enriched | Growth hormone secretagogue (ghrelin) receptor |
| *Gpx3* | EW-Enriched | Glutathione peroxidase 3 |
| *Lepr* | EW-Enriched | Leptin receptor |
| *Ndn* | EW-Enriched | Necdin |
| *Nenf* | EW-Enriched | Neuron-derived neurotrophic factor |
| *Neto1* | EW-Enriched | Neuropilin and tolloid-like 1 |
| *Nucb2* | EW-Enriched | Nucleobindin-2 (Nesfatin-1) |
| *Pcsk1* | EW-Enriched | Proprotein convertase subtilisin/kexin type 1 |
| *Peg3* | EW-Enriched | Paternally-expressed gene 10 |
| *Peg10* | EW-Enriched | Paternally-expressed gene 3 |
| *Postn* | EW-Enriched | Periostin, osteoblast specific factor |
| *Prmt2* | EW-Enriched | Protein arginine methyltransferase 2 |
| *Ptprn* | EW-Enriched | Protein tyrosine phosphatase, receptor type N |
| *Scg2* | EW-Enriched | Secretogranin-2, secretoneurin |
| *Sncg* | EW-Enriched | Gamma-synuclein |
| *Ucn* | EW-Enriched | Urocortin-1 |
| *Egr1* | ITFs | Early growth response 1 |
| *Fos* | ITFs | FBJ osteosarcoma oncogene |
| *Fosb* | ITFs | FBJ osteosarcoma oncogene B |
| *Th* | DA-Related | Tyrosine hydroxylase |
| *Ddc* | DA-Related | Dopamine decarboxylase |
| *Slc6a3* | DA-Related | Dopamine reuptake transporter |
| *Drd1a* | DA-Related | Dopamine receptor 1 |
| *Drd2* | DA-Related | Dopamine receptor 2 |
| *Drd3* | DA-Related | Dopamine receptor 3 |
| *Drd4* | DA-Related | Dopamine receptor 4 |
| *Drd5* | DA-Related | Dopamine receptor 5 |
| *Crhr1* | CRF-Related | CRF receptor 1 |
| *Crhr2* | CRF-Related | CRF receptor 2 |
| *Crhbp* | CRF-Related | CRF binding protein |

**Table S3: Effects of Long-Term Alcohol Drinking on EWcp Gene Expression**

Seventeen of 37 genes of interest were significantly upregulated in Alcohol vs. Naïve mice (*p* < 0.05, 1.4-fold to 2.9-fold). No genes were significantly downregulated in Alcohol vs. Naïve mice.

| **Gene** | ***p*** | **Fold** | **Gene Name and Information** |
| --- | --- | --- | --- |
| *Adcyap1* | .0052 | 1.6942 | Pituitary AC-activating peptide (PACAP; stress neuropeptide) |
| *Cds2* | .0028 | 1.7994 | Phosphatidate cytidylyltransferase 2 |
| *Gabrq* | .0236  .01 | 1.5634 | GABA-A receptor subunit theta |
| *Gpx3* | .0134 | 1.8415 | Glutathione peroxidase 3 |
| *Ndn* | .0040 | 1.7512 | Necdin (Deleted in Prader-Willi syndrome; imprinted) |
| *Nenf* | .0393 | 1.4533 | Neuron-derived neurotrophic factor |
| *Nucb2* | .0166 | 1.6309 | Nucleobindin-2 (Nesfatin-1; stress neuropeptide) |
| *Pcsk1* | .0005 | 1.9052 | Proprotein convertase 1 (Cleaves long-form peptides) |
| *Peg3* | .0055 | 1.8870 | Paternally-expressed gene 3 (Imprinted) |
| *Peg10* | .0165 | 1.8238 | Paternally-expressed gene 10 (Imprinted) |
| *Postn* | .0258 | 2.2438 | Periostin (Ligand for integrins that support cell migration) |
| *Prmt2* | .0131 | 1.5785 | Protein arginine methyltransferase 2 (Methylation; ER-α) |
| *Scg2* | .0001 | 2.0169 | Secretogranin-2 (Packages neuropeptide vesicles) |
| *Ucn* | .0183 | 2.0261 | Urocortin-1 (Stress neuropeptide) |
| *Drd5* | .0072 | 2.9283 | Dopamine D5 receptor (D1-like, Gs-coupled) |
| *Crhbp* | .0022 | 1.7433 | CRF binding protein (Interacts with Ucn1) |
| *Crhr1* | .0476 | 1.8605 | CRF type-1 receptor (Interacts with Ucn1) |

**SUPPLEMENTAL REFERENCES**

Helms, C. M., Reeves, J. M., & Mitchell, S. H. (2006). Impact of strain and d-amphetamine on impulsivity (delay discounting) in inbred mice. Psychopharmacology, 188, 144-151. PMID: 16915383

Mitchell, S. H. (2014). Assessing delay discounting in mice. Current Protocols in Neuroscience, 66: 8.30.1–8.30.12. doi:10.1002/0471142301.ns0830s66. PMID: 24510779 [PubMed - in process]

Wilhelm, C. J., & Mitchell, S. H. (2009). Strain differences in delay discounting in inbred rats. Genes, Brain & Behavior, 8, 426-434. PMCID: PMC2825220

Wilhelm, C. J., & Mitchell, S. H. (2012). Acute ethanol does not always affect delay discounting in rats selected to prefer or avoid ethanol. Alcohol and Alcoholism, 47, 518-24. PMCID: PMC3500854
