# Supplementary figures and images for: Control of chronic excessive alcohol drinking by genetic manipulation of the Edinger–Westphal nucleus urocortin-1 neuropeptide system
Source: Transl Psychiatry. 2017 Jan 31;7(1):e1021–. doi: 10.1038/tp.2016.293 (PMC5299395; doi:10.1038/tp.2016.293)

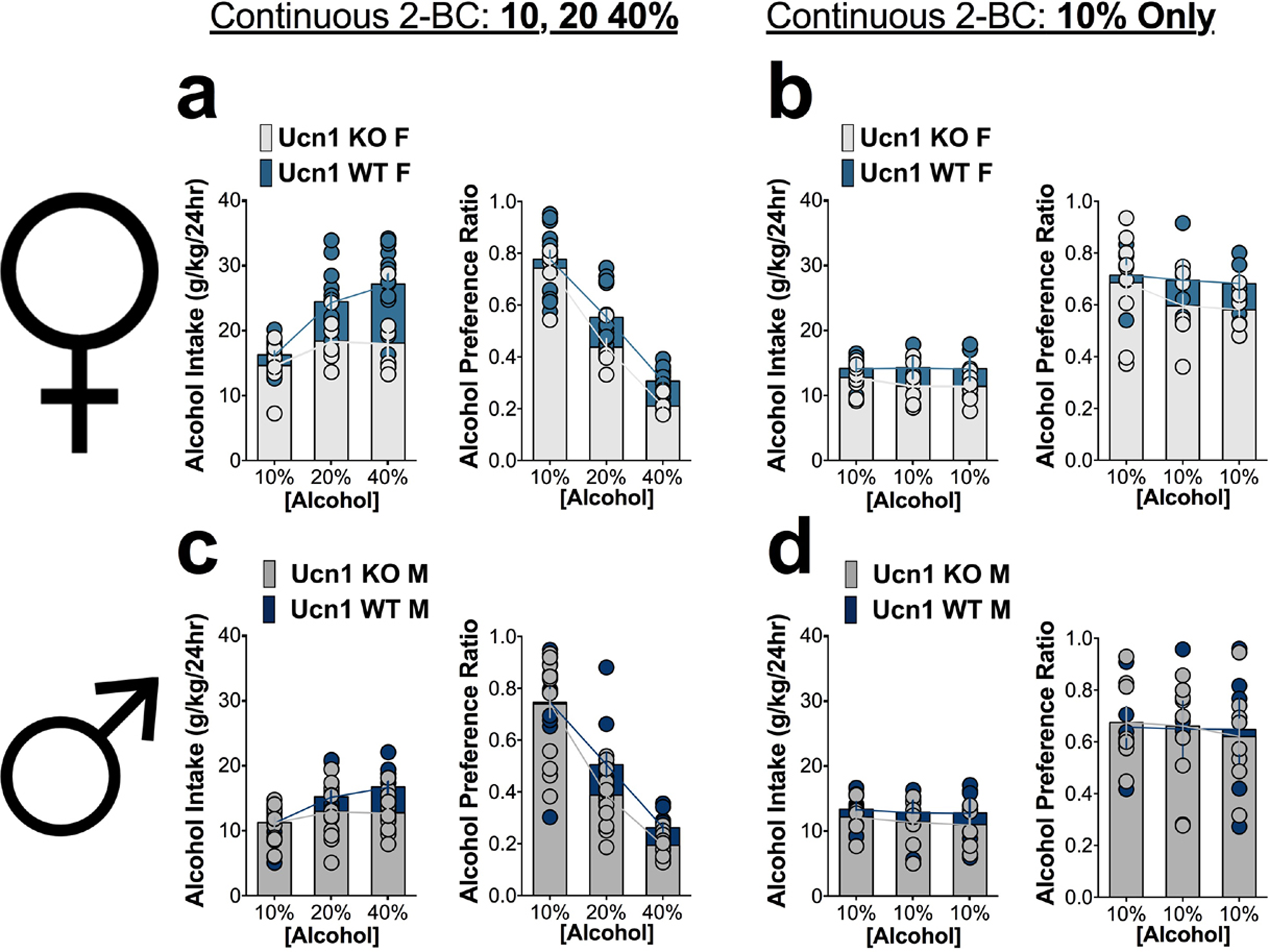

Supplement: Supplementary Figure S1 [file tp2016293x2.tif]

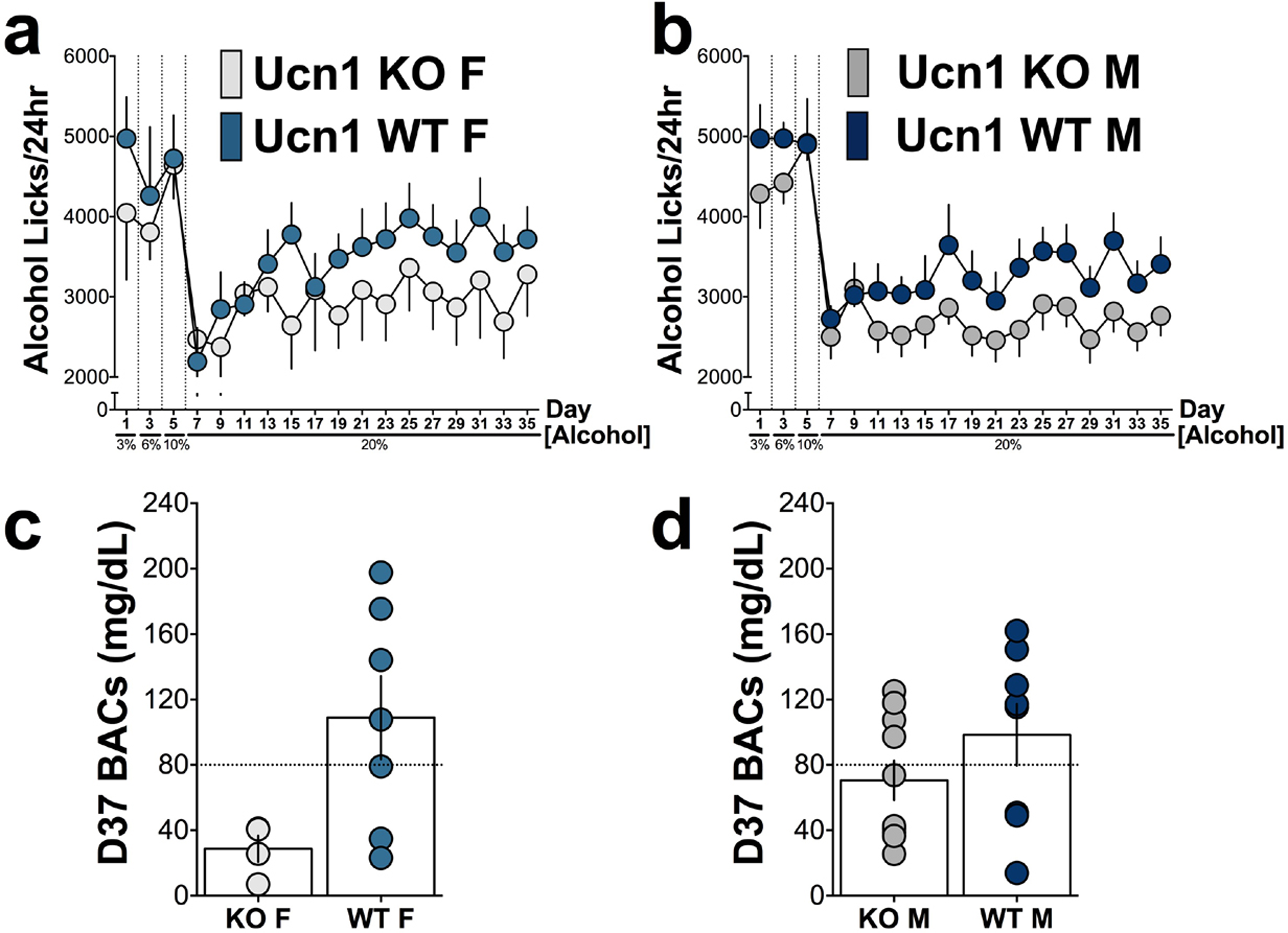

Supplement: Supplementary Figure S2 [file tp2016293x3.tif]

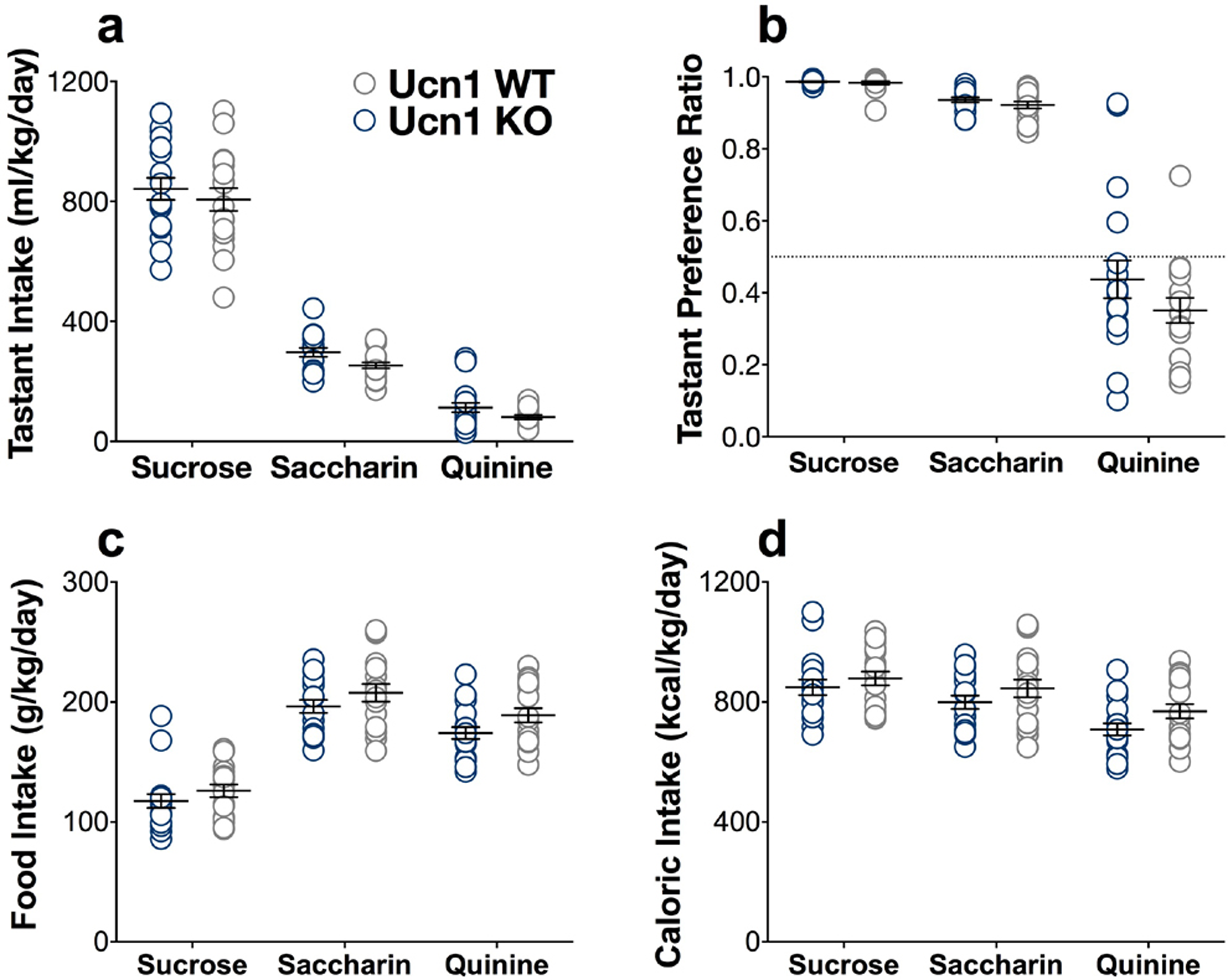

Supplement: Supplementary Figure S3 [file tp2016293x4.tif]

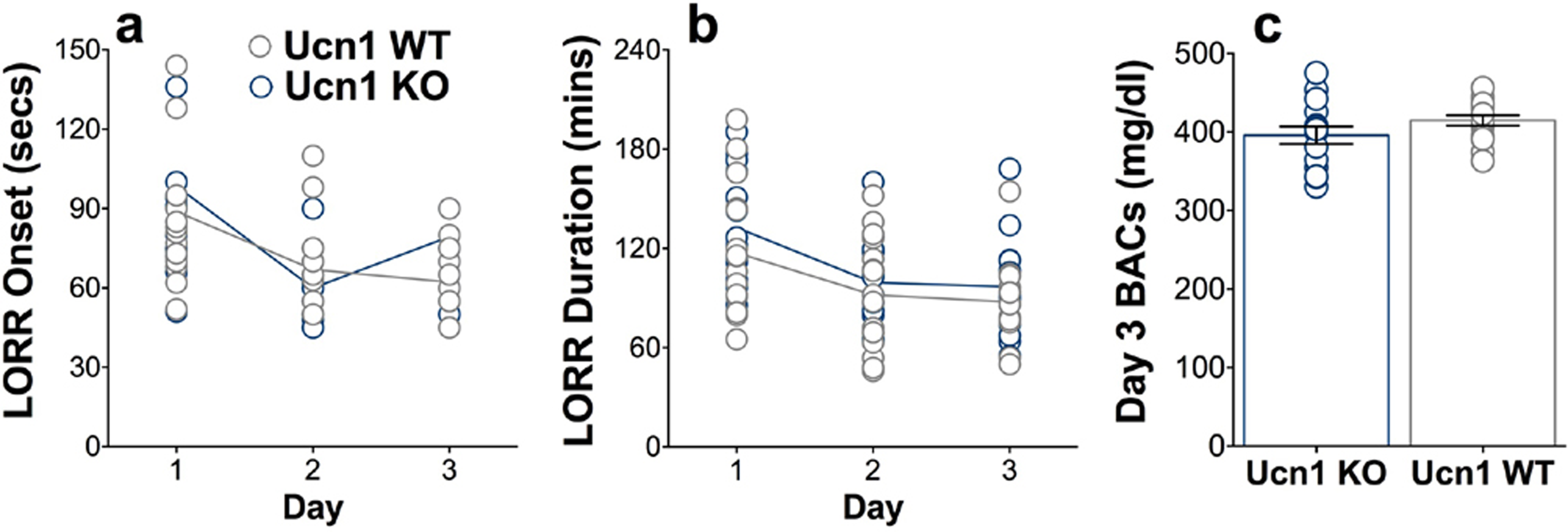

Supplement: Supplementary Figure S4 [file tp2016293x5.tif]

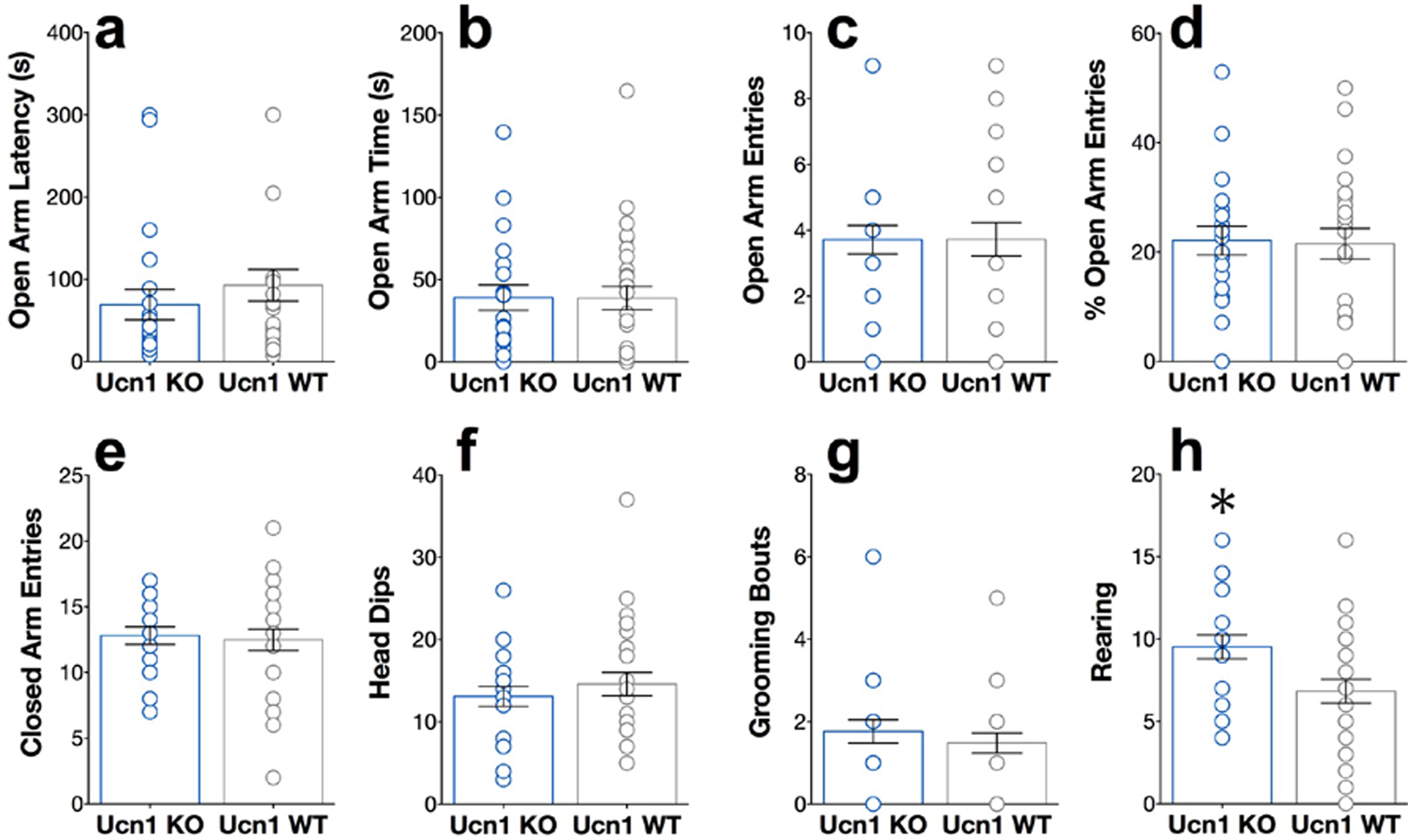

Supplement: Supplementary Figure S5 [file tp2016293x6.tif]

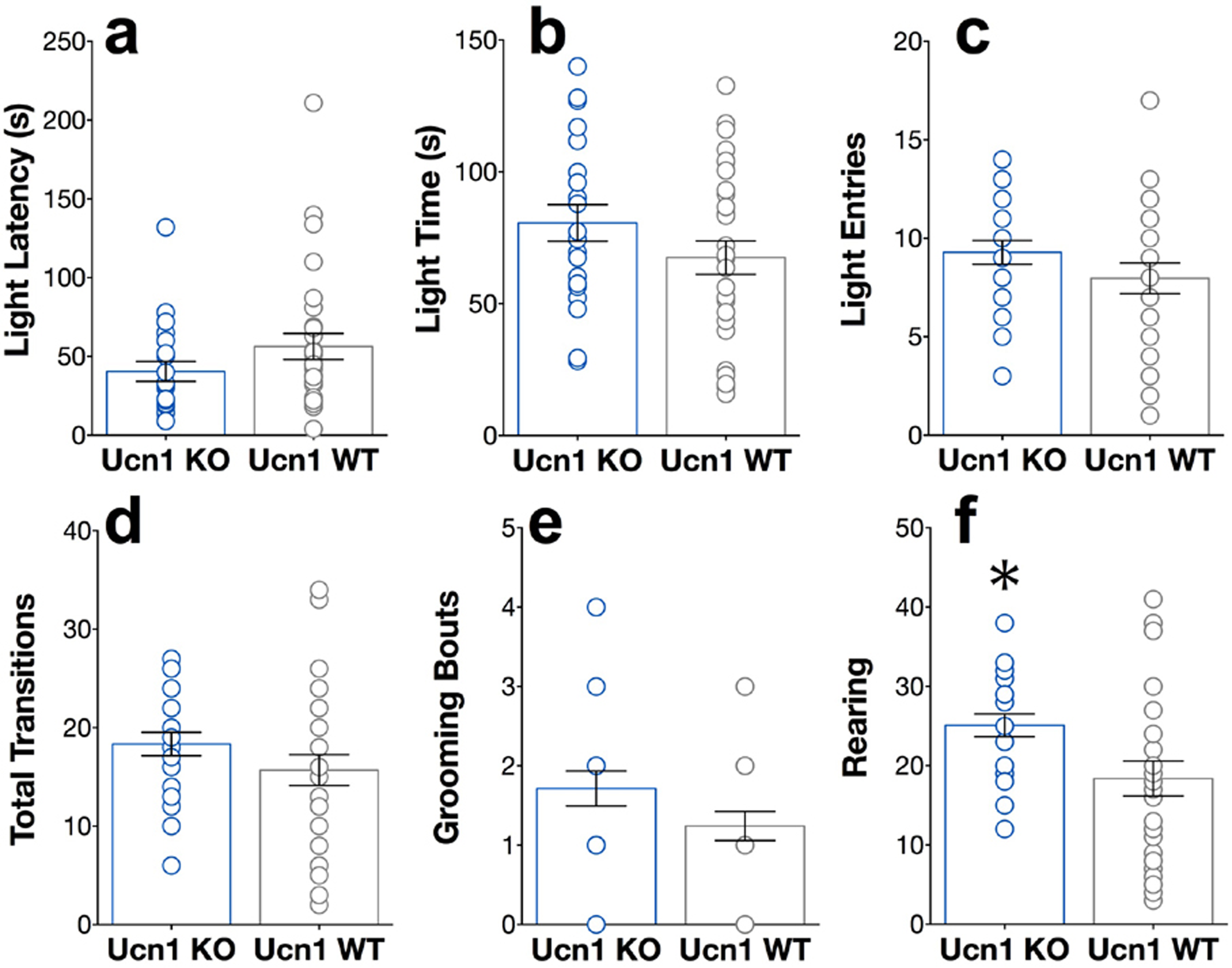

Supplement: Supplementary Figure S6 [file tp2016293x7.tif]

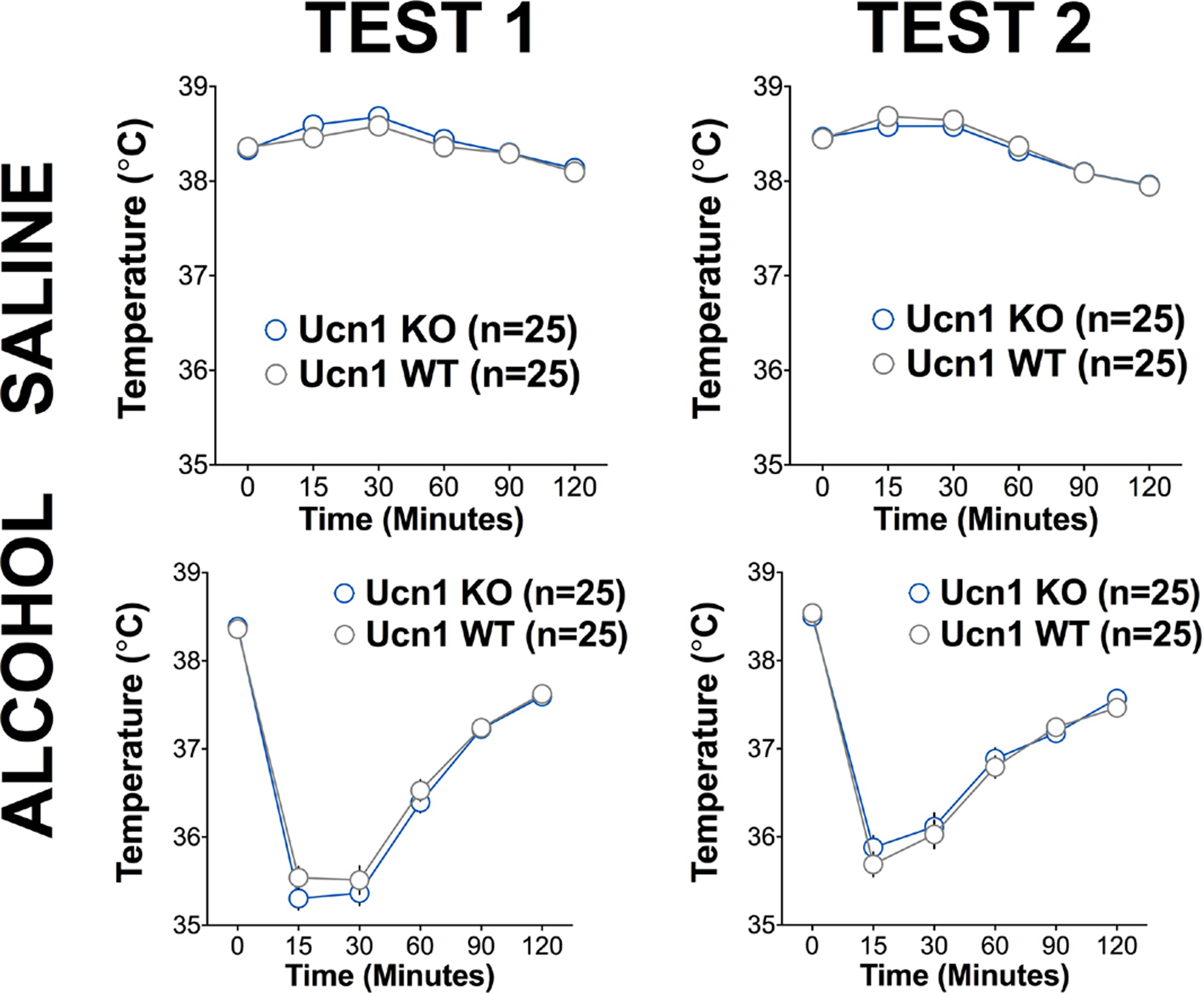

Supplement: Supplementary Figure S7 [file tp2016293x8.tif]

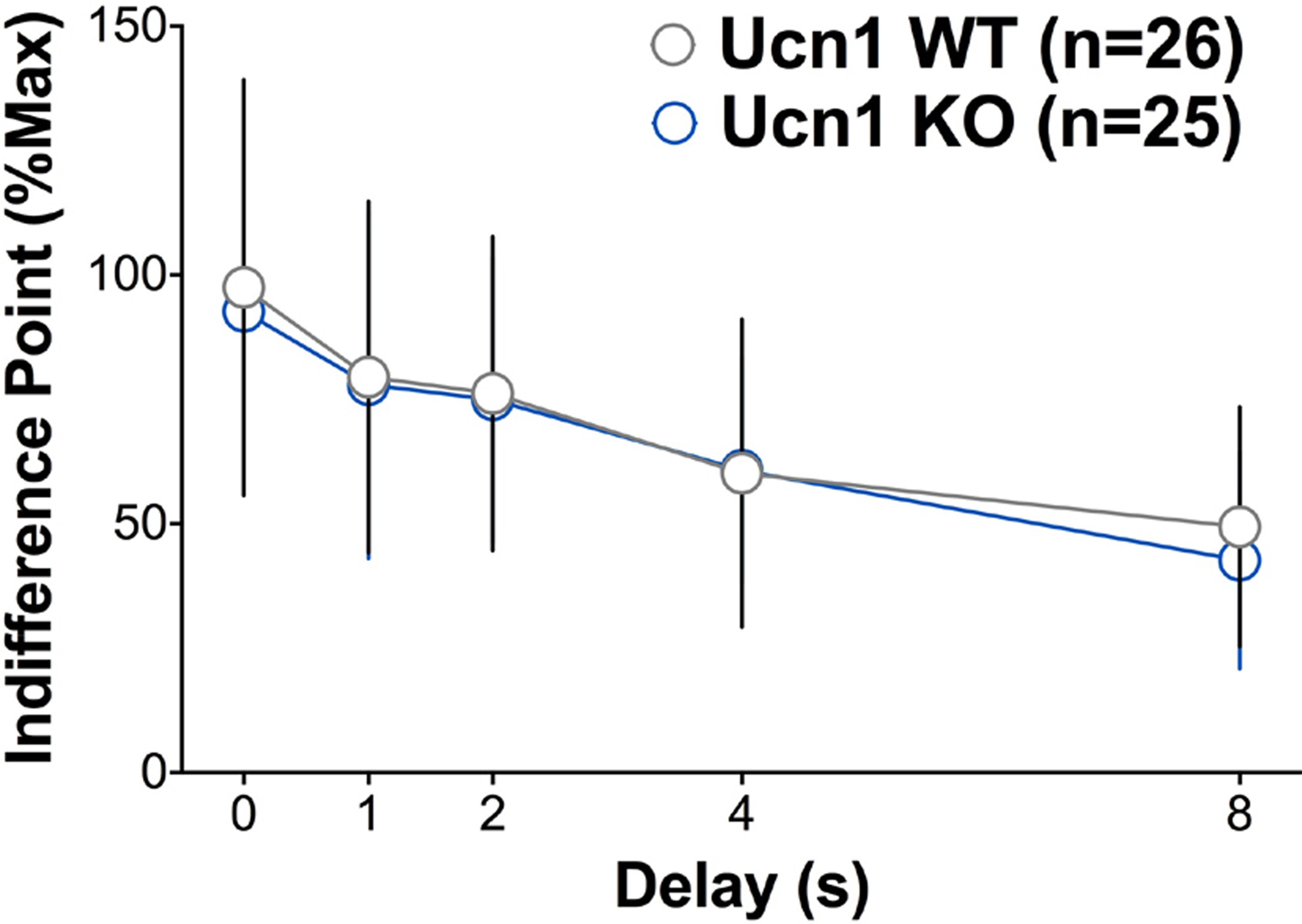

Supplement: Supplementary Figure S8 [file tp2016293x9.tif]

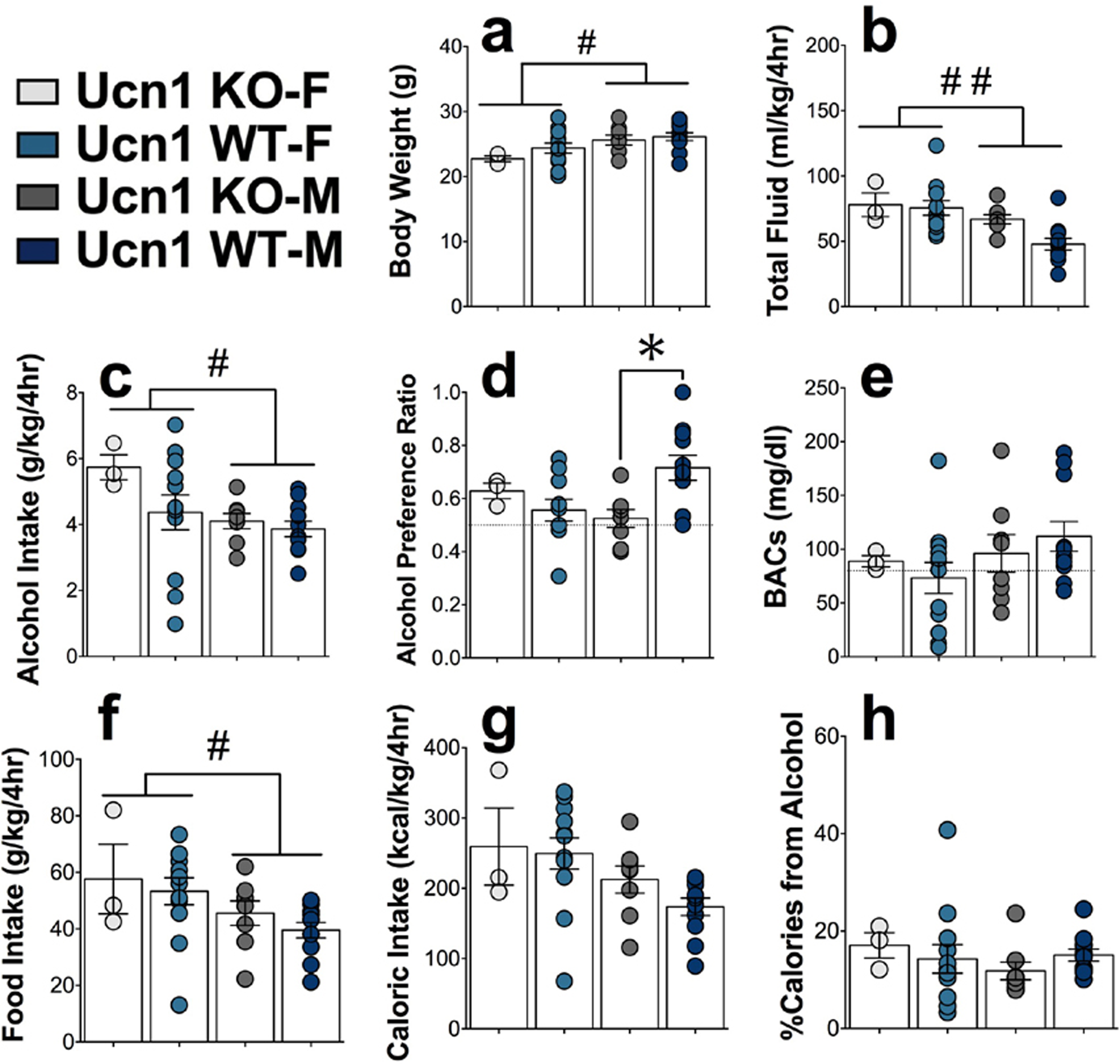

Supplement: Supplementary Figure S9 [file tp2016293x10.tif]

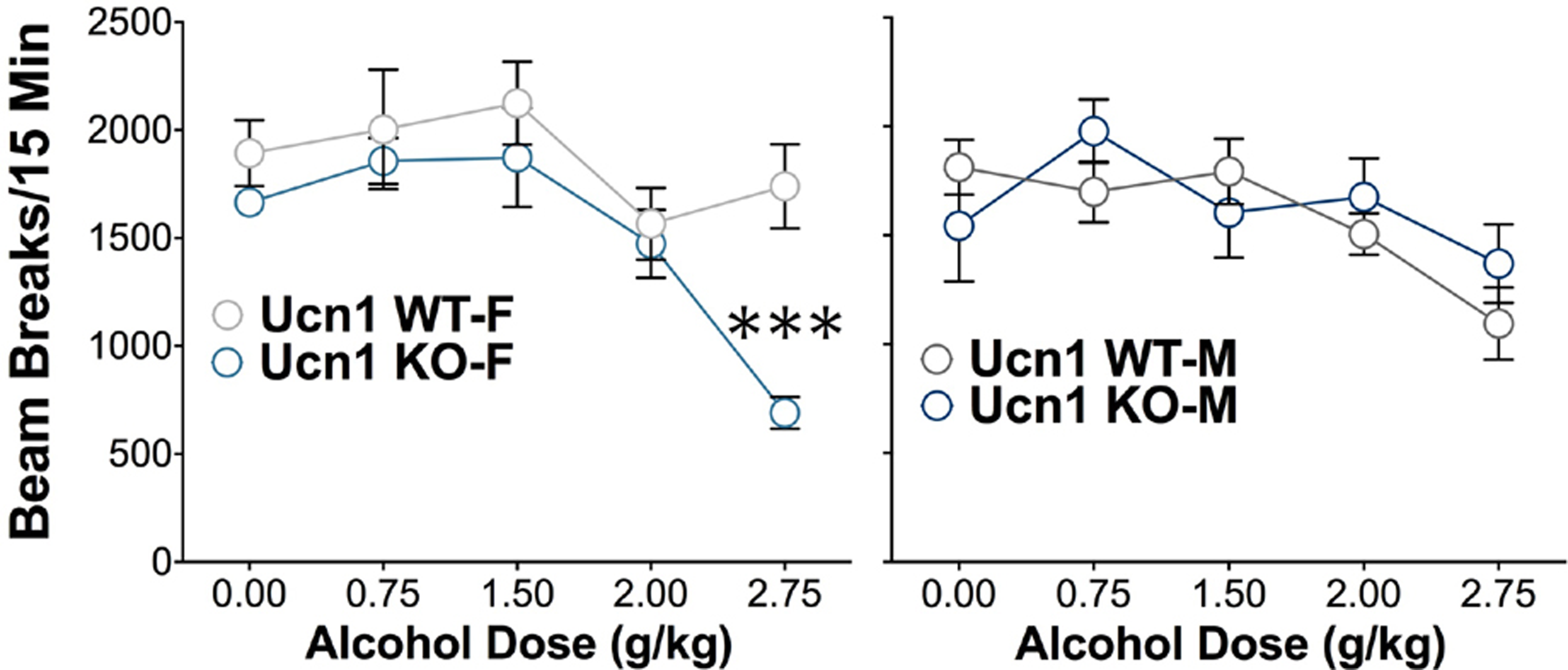

Supplement: Supplementary Figure S10 [file tp2016293x11.tif]
